# Perioperative acute kidney injury

**DOI:** 10.1186/2047-0525-1-6

**Published:** 2012-07-04

**Authors:** Stacey Calvert, Andrew Shaw

**Affiliations:** 1 St George’s Hospital, London, SW17 0QT, UK; 2 Dept of Anesthesiology and Critical Care Medicine, Duke University Medical Center/Durham VAMC, Durham, USA

**Keywords:** Acute kidney injury, Biomarkers, Perioperative, Pharmacological interventions, Risk stratification

## Abstract

Acute kidney injury (AKI) is a serious complication in the perioperative period, and is consistently associated with increased rates of mortality and morbidity. Two major consensus definitions have been developed in the last decade that allow for easier comparison of trial evidence. Risk factors have been identified in both cardiac and general surgery and there is an evolving role for novel biomarkers. Despite this, there has been no real change in outcomes and the mainstay of treatment remains preventive with no clear evidence supporting any therapeutic intervention as yet. This review focuses on definition, risk factors, the emerging role of biomarkers and subsequent management of AKI in the perioperative period, taking into account new and emerging strategies.

## Review

### Introduction

Acute kidney injury (AKI) occurs in 1% to 5% of all hospital admissions, and in the perioperative period has serious implications, being consistently associated with (unacceptably) high mortality, morbidity and a more complicated hospital course with associated cost implications. This is particularly the case when renal replacement therapy (RRT) is required [1-22]. It is widely recognized that AKI requiring dialysis is an independent risk factor for death [1-3]; more recently, however, even minimal increases in serum creatinine have been associated with an increase in both short and long-term mortality, regardless of whether partial or full recovery of renal function has occurred at the time of discharge [4-11]. This risk of death is independent from other postoperative complications and co-morbidities [7-9]. AKI is related to the subsequent development and progression of chronic kidney disease (CKD) and the need for future dialysis, most notably in those with a degree of pre-existing renal impairment [11-15], but also in those who have apparent recovery following an episode of AKI [7]. Despite an increase in our knowledge of AKI and advances in other relevant areas over the last two decades (including intensive care, delivery of dialysis and surgical techniques), there have been no significant changes in these outcomes [12,15-17]. As such, identification of risk factors, close monitoring of renal function and early adoption of both preventive measures and treatments remain important considerations for those taking care of perioperative patients who are likely to develop AKI.

### Incidence

Surgery remains a leading cause of AKI in hospitalized patients (the incidence ranges from 18% to 47% depending on the definition used) [17,18]. This has been best researched in the cardiac surgery setting where it has been shown that up to 15% of patients exposed to cardiopulmonary bypass (CPB) will develop AKI, with 2% requiring RRT [23]. Depending on the criteria used to define AKI and the postoperative period studied, mortality ranges from 1% to 30% [5,24] although this is consistently higher, approaching 80%, if RRT is required [2,17,24]. AKI is not limited to cardiac surgery although its incidence outside of this setting is often underappreciated. Kheterpal *et al.* demonstrated that in patients without pre-existing renal disease, approximately 1% of major non-cardiac surgery was complicated by AKI, with an eight-fold increase in 30-day mortality [20,23]. This incidence is comparable to other notable postoperative complications including major adverse cardiac events (MACE) and venous thromboembolism [23].

In the intensive care setting, the Beginning and Ending Supportive Therapy for the Kidney (BEST Kidney) investigators confirmed major surgery as the second leading cause of AKI (in 34%) in this cohort of patients, with overall hospital mortality of 60.3% [1]. Analysis of data from the United Kingdom Intensive Care National Audit and Research Centre Case Mix program supports this, showing surgical admissions accounted for 16.4% of admissions with severe AKI in the first 24 hours (with elective and emergency cases accounting for 5.6% and 10.8%, respectively). In that study, defining severe AKI as creatinine >300 μmol/l and/or urea >40 mmol/l has restricted the patient cohort and potentially, therefore, may limit its generalizability [25]. Elsewhere, it has been reported that one third of patients with AKI require a critical care admission at some point in their care [14].

### Definition

Although AKI has been the focus of much research over the past decades, lack of a consensus definition has been a major factor hampering clinical research and comparison of trial data [1,12,13,22,26,27]. There are now two major classifications of AKI in use. The Acute Dialysis Quality Initiative (ADQI) Group introduced the RIFLE (Risk, Injury, Failure, Loss and End-stage) classification system in 2004, which defines three grades of severity and two outcomes, in an effort to standardize the definition [7,12,28,29]. This has subsequently been validated in a number of studies [7,29-35]. The Acute Kidney Injury Network (AKIN) group proposed refinements to this criteria, outlining AKI as abrupt (occurring within 48 hours) and using a smaller change in serum creatinine from baseline in patients who are optimally hydrated to define AKI [12,28,29], following recognition of emerging evidence demonstrating the clinical importance of small increases in serum creatinine [5-9]. No clear advantages between these criteria have been demonstrated and despite these recommendations, definitions of AKI continue to vary [29]. The Kidney Disease: Improving Global Outcomes (KDIGO) workgroup has recently reviewed these criteria and published a single definition for use in both clinical practice and research. AKI is defined when any of the following three criteria are met; an increase in serum creatinine by 50% in seven days, an increase in serum creatinine > 0.3 mg/dL in 48 hours or oliguria. The severity is staged according the criteria outlined in Table 1[36].

**Table 1:** **Classification of acute kidney injury by RIFLE, AKIN and KIDGO criteria**[12,28,36]

**Stage**	**Glomerular filtration rate (GFR) criteria**	**Urine output criteria**
*RIFLE classification*		
**Risk**	Serum creatinine increased x 1.5 or GFR decrease >25%	<0.5 ml/kg/hr for ≥ 6 hours
**Injury**	Serum creatinine increased x 2 or GFR decrease >50%	<0.5 ml/kg/hr for ≥ 12 hours
**Failure**	Serum creatinine increased x 3 or GFR decrease ≥ 75% or an absolute serum creatinine ≥ 354 μmol/L with an acute rise ≥ 4 μmol/L	<0.3 ml/kg/hr for ≥ 24 hours or anuria for ≥12 hours
**Loss**	Persistent AKI, requiring RRT for > 4 weeks	
**End-stage kidney disease**	Requiring dialysis > 3 months	
*AKIN classification*		
**Stage 1**	Serum creatinine increased ≥26.2 μmol/L or x 0.5 to 2 baseline	<0.5 ml/kg/hr for ≥ 6hours
**Stage 2**	Serum creatinine increased x 2 to 3 baseline	<0.5 ml/kg/hr for ≥ 12 hours
**Stage 3**	Serum creatinine increased > x 3 baseline or serum creatinine ≥ 354 μmol/L with an acute rise ≥ 44 μmol/L or initiation of RRT	<0.3 ml/kg/hr for ≥ 24 hours or anuria for ≥12 hours
*KDIGO classification*		
**Stage 1**	Serum creatinine increased x 1.5 to 1.9 baseline or by ≥ 26.2 μmol/L	<0.5 ml/kg/hr for 6 to 12 hours
**Stage 2**	Serum creatinine increased x 2 to 2.9 baseline	<0.5 ml/kg/hr for ≥ 12 hours
**Stage 3**	Serum creatinine increased > x 3 baseline or serum creatinine ≥ 354 μmol/L with an acute rise ≥ 44 μmol/L or initiation of RRT	<0.3 ml/kg/hr for ≥ 24 hours or anuria for ≥12 hours

*AKIN*, Acute Kidney Injury Network; *KDIGO*, Kidney Disease: Improving Global Outcomes.

Recognition is often still delayed and more recently, the role of electronic reporting systems has been successfully tested in the UK with the aim of alerting clinicians early to the presence of AKI, appreciating the impact of small increases in creatinine from baseline that previously may have been considered as fluctuations remaining within the normal range. In turn, this should allow for timely intervention and improved overall patient care [37].

RIFLE, AKIN and KDIGO all diagnose AKI according to serum creatinine and urine output as outlined in Table 1. This, however, is not without its limitations, as serum creatinine is neither sensitive nor specific, tending to represent a functional change rather than being a true marker of kidney injury and is well known to be affected by multiple factors including age, ethnicity, gender, muscle mass, total body volume, medications and protein intake [16,38]. Given that a reduction in glomerular filtration rate (GFR) greater than 50% can occur before this is reflected in serum creatinine [16,39,40], the ability to detect AKI prior to a change in serum creatinine would represent a significant advance in the management of AKI. As such, the American Society of Nephrology set identification and characterization of biomarkers for AKI as a key research area in 2005 [41].

### Risk factors

There have been a number of studies investigating the risk factors associated with the development of AKI, from which several factors, both patient and procedure related, have been consistently associated in both cardiac and non-cardiac surgery (Table 2) [3,20,23,24,42-45]. Patient related factors are often more strongly associated with postoperative mortality than surgical factors. These include age, hypertension, diabetes mellitus, cardiac failure, peripheral vascular disease, cerebrovascular disease and pre-existing chronic kidney disease [3,23,24,42-44]. Perhaps the most important of these is the latter, with rates of AKI requiring dialysis approaching 30% in patients with pre-existing kidney disease undergoing cardiac surgery [7,17,24,42-44]. That said, there remain risk factors specific to certain types of surgery which are associated with postoperative AKI, including prolonged CPB time, combined valve and coronary artery bypass graft (CABG) surgery, increased aortic cross-clamp time during vascular surgery and increased intra-abdominal pressure in major abdominal surgery [2,17,46]. Unsurprisingly, many of these risk factors are associated with either poor renal perfusion or decreased renal reserve and few are correctable prior to surgery.

**Table 2:** Factors associated with the development of AKI

**Patient related factors**	**Surgical factors**
Age	Duration of surgery
Hypertension	Intra-peritoneal surgery
Diabetes Mellitus	Length of CPB
Chronic Obstructive Pulmonary Disease	Cross clamp time
LVF, EF <40%	Hemolysis (cardiac surgery)
Chronic kidney disease	Hemodilution (cardiac surgery)
Emergency surgery	Use of IABP (cardiac surgery)
Sepsis	
Peripheral vascular disease	
Cerebrovascular disease	
Ascites	

*EF*, ejection fraction; *IABP*, Intra-aortic balloon pump; *LVF*, left ventricular function.

Integral to improving outcomes, however, is the ability to identify high risk patients, not only allowing for earlier intervention and optimal subsequent management, but also identification of cohorts of patients in which new treatments can be studied. Several groups have, therefore, sought to develop risk stratification indexes in both cardiac and general surgery [23,24,42,45,47]. Kheterphal *et al.* developed a General Surgery AKI Risk Index after evaluating almost 76,000 general surgical patients, which also included a validation sample. A score is given for each patient, based on nine separate preoperative risk factors, following which patients are categorized into one of five classes. Class I (determined by having zero to two risk factors) has an incidence of AKI of 0.2%; in contrast to Class V (>6 risk factors) which confers an AKI risk of 9.5% [23]. Although useful in highlighting risk factors, further validation over multiple centers is crucial, with similar single center risk scoring systems post-cardiac surgery having been shown to underestimate the true incidence of AKI, despite taking into account demographic variation [47].

### Novel biomarkers

An ideal biomarker would be highly sensitive and specific for AKI, responding consistently and rapidly to injury, with normal ranges for age, race and gender established and levels that correlate to severity as well as having biological stability and a reliable, quick and cost effective assay for detection [48,49]. It would also be useful to determine the extent of interpersonal variation attributable to genetic factors and the impact of confounding clinical factors [48]. The area under the receiver-operating characteristic curve (AUC_ROC_) is used to assess the performance of a diagnostic biomarker, with a value greater than 0.75 demonstrating good discriminatory value and greater than 0.90 demonstrating excellent discrimination.

A current challenge is that novel biomarkers are being compared to serum creatinine as the ‘gold standard’ when it is the very weakness of serum creatinine as a sensitive and specific marker that prompted research into this area. Indeed, many authors have made this exact point in their opening statements [50]. More than 20 different biomarkers have been identified in recent years, predominantly in studies of post-cardiac surgery. However, most current focus is on neutrophil gelatinase-associated lipocalin (NGAL), kidney injury molecule − 1 (KIM-1), IL-18 and cystatin-C. At present these remain experimental and need validation in larger studies prior to transition into clinical practice [38]. It is highly likely that several other biomarkers will also be introduced into clinical practice over the next few years.

### NGAL

NGAL has generated significant interest in recent years, particularly in AKI following cardiac surgery, although its use is not restricted to this cohort of patients [39,51-53]. In patients with normal renal function, NGAL is almost undetectable in either urine or plasma, yet animal studies clearly demonstrated that NGAL is markedly upregulated early following ischemic injury [54]. In subsequent clinical studies, urinary NGAL has been shown to be both sensitive and specific in predicting postoperative AKI in pediatric patients undergoing cardiac surgery [43,51,52]. Similarly, plasma NGAL measured at two hours post-CPB correlated strongly with severity and duration of AKI, with an AUC_ROC_ of 0.96, sensitivity of 0.84 and specificity of 0.94 [39]. In an adult population, this result has been less consistent [51,52] with Wagener *et al.* demonstrating an AUC_ROC_ of 0.61 and sensitivity of 0.39 in urinary NGAL measured 18 hours post-surgery [52]. Likewise, raised plasma NGAL levels have been clearly demonstrated in AKI following CPB surgery, however again with a low sensitivity thereby limiting its use as a single biomarker in the prediction of AKI [55]. It has been proposed that this poor sensitivity may be in part due to the current limitations of defining AKI using serum creatinine [52], although it should also be noted that patients who develop AKI also tend to have a longer CPB time. Although this has been clearly demonstrated to be a risk factor, it also raises the possibility that NGAL (particularly plasma NGAL) could actually reflect length of CPB/degree of inflammation versus degree of kidney injury [39]. Many of these studies have, however, excluded patients with pre-existing renal dysfunction. A *post-hoc* subgroup analysis has attempted to address this and although these results must be interpreted with a degree of caution, they do show that the use of urinary NGAL is significantly influenced by pre-existing renal function, with no clear relationship between postoperative urinary NGAL and the development of AKI in patients with a GFR < 60 ml/minute [56]. This suggests that the relationship between NGAL and AKI is complex and is likely to be different in the setting of CKD [57].

### KIM-1

KIM-1 is a type 1 transmembrane glycoprotein, undetectable in normal kidney tissue, which has been shown to be markedly upregulated following injury secondary to ischemia and nephrotoxins in a variety of both animal and human studies, with a soluble form readily detectable in the urine [58]. Early human studies demonstrated a clear increase in KIM-1 protein expression at biopsy that correlated with high urinary levels, detectable prior to cast formation, following ischemic injury [58]. Since then, KIM-1 has been shown to be a highly sensitive marker for AKI in patients undergoing cardiac surgery [59] and, alongside another urinary biomarker, N-acetyl-β-(D)-glucosaminidase, high levels have been associated with adverse outcomes including the need for renal replacement therapy and death [60].

### IL-18

The cytokine IL-18 has also been shown to be an early biomarker for AKI in a variety of clinical situations, including in patients with CKD [61-65]. Post-CPB surgery, urinary IL-18 was detectable four to six hours post-surgery, peaking at 12 hours with an AUC_ROC_ of 0.75 and remaining elevated over the next 24 to 28 hours (AUC_ROC_ at 24 hours 0.75) [61]. In addition, there is a correlation between peak levels and increased severity of AKI and mortality [63,64]. Unsurprisingly, given the role of IL-18 as a pro-inflammatory cytokine, levels are higher in cohorts of patients with sepsis than in those without [64].

### Cystatin C

Cystatin C is a cysteine protease inhibitor produced by all nucleated cells. Given that it is freely filtered by the glomerulus, undergoes almost complete tubular reabsorption and is not secreted by renal tubules, it is desirable as a marker of GFR [40,66-68]. However, serum cystatin C levels have been shown to be affected by the use of steroids, thyroid dysfunction, age, gender and CRP independent of GFR [40,66,67]. A prospective study looking at 72 patients undergoing cardiac surgery demonstrated no clear association between AKI and plasma cystatin C although an early and persistent increase in urinary cystatin C was associated with AKI, and the level excreted correlated with the severity of AKI. This suggests that in this cohort of patients, urinary cystatin C may be more useful [67].

Importantly, many studies to date have excluded patients with CKD, who have been consistently demonstrated to be at high risk for AKI in the perioperative period [20] and these biomarkers must therefore first be characterized over a range of baseline values, with more information required to identify and explain clinical factors that may confound their performance in the perioperative period [38-40,51-57,67]. It is unlikely that any single biomarker would be sufficient for accurate diagnosis and risk stratification of AKI but rather that the way forward would be to develop a panel of biomarkers which, used in conjunction, would allow for assessment of disease severity and risk alongside earlier diagnosis [40,49,59,61]. This faces its own challenges and as yet there is insufficient information as to which combinations to recommend for use. More work is clearly needed in this area, with the ultimate aim being earlier recognition of AKI, thereby allowing for progress to be made in its subsequent treatment.

### Pathophysiology of AKI

Etiologically, AKI is divided into pre-renal, intrinsic or renal, and post-renal causes, in surgery representing 30% to 60%, 20% to 40% and 1% to 10% of cases, respectively [17] (Table 3). Renal hypoperfusion is often the initial insult in perioperative AKI, which importantly can lead to a reduction in medullary blood flow [17,46,69]. The outer medulla with its high metabolic demands (medullary oxygen extraction approaches 90%) is particularly vulnerable to both hypoperfusion and hypoxia, both in patients with known CKD whose underlying reserve is reduced but also in patients with normal preoperative renal function [69-71]. Interestingly, in acute respiratory distress syndrome (ARDS) it is increasingly recognized that the disease process, for example, pulmonary versus extra-pulmonary causes, impacts the course of the disease and whether the same could be said for AKI remains to be seen [72].

**Table 3:** Summary of causes of AKI defined etiologically

**Pre-renal**	**Intrinsic renal disease**	**Post-renal**
Hypovolemia, for example, hemorrhage, diarrhea, vomiting	Ischemia from prolonged hypoperfusion	Obstructive causes, for example, prostatic hypertrophy, renal stones, urethral strictures, pelvic masses
Hypotension, for example, sepsis	Glomerular disease, for example, glomerulonephritis, TTP, DIC	
Low cardiac output state, for example, CCF, cardiac tamponade	Nephrotoxins, for example, aminoglycosides, NSAIDs, radiological contrast	
Impaired renal autoregulation, for example, renal artery stenosis, ACEi/ARB/NSAIDs	Metabolic abnormalities, for example, hypercalcemia	
	Rhabdomyolysis, for example, crush injuries, burns	

*ACEi*, angiotensin converting enzyme inhibitor; *ARB*, angiotensin recreptor blocker; *CCF*, congestive cardiac failure; *DIC*, disseminated intravascular coagulation; *TTP*, thrombotic thrombocytopenic purpura.

Animal models have been developed, predominantly based on ischemia-reperfusion injury or drug-induced injury, which have significantly improved our understanding of AKI, especially with regard to the role of inflammation. This is thought to be especially important in AKI associated with CPB surgery [73]. In clinical practice, ischemia-reperfusion injury can occur secondary to either general hypoperfusion or specific actions, for example, cross-clamping of the aorta in vascular surgery. Interventions that are beneficial in animal models, however, have not yet been shown to be effective in clinical practice [74,75].

Histologically, there is still a paucity of information available, in part due to the invasive nature of renal biopsies that are often not undertaken in patients in whom AKI is presumed secondary to pre-renal factors [74]. In biopsies that have been obtained, and from post-mortem findings, there is a clear disparity seen between the clinical scenario and the pathological findings [74]. This, in turn, supports the concept of cytopathic hypoxia leading to cellular shutdown versus cell necrosis or apoptosis [75].

### Management of AKI

The goals in management of AKI include preservation of existing renal function as well as prevention of acute complications (hyperkalemia, acidosis, volume overload) and the need for long-term renal replacement therapy. Avoidance of AKI remains the cornerstone of management while research continues into effective treatment options.

### Preventive measures

#### Fluids and goal directed therapy

Maintenance of normal renal perfusion is perhaps the most important prophylactic measure, with 80% of patients experiencing postoperative AKI having an episode of hemodynamic instability in the perioperative period [17,46]. The use of fluids in this period is therefore vital although this should be approached with caution as there are equally important recognized postoperative complications associated with excess fluid including poor wound healing and increased duration of mechanical ventilation [76,77]. There is increasing evidence that a positive fluid balance in both surgical and critical care patients is associated with an increase in intra-abdominal hypertension which, in turn, has a detrimental effect on renal function [77-79]. Furthermore, hyperchloremia is often associated with over-zealous fluid resuscitation with 0.9% saline and has been associated with a decrease in renal blood flow [77]. Importantly, studies comparing conservative versus liberal fluid strategies have not seen an increase in the incidence in AKI or an increased need for RRT in the conservative arms [77].

There has, however, been no randomized controlled trial (RCT) directed at addressing the role of fluid hydration in the prevention of AKI in surgical patients [80] and this task often falls to junior members of the team. A targeted approach with titration to specified end-points may in fact be more appropriate [77,81].

Goal directed therapy (GDT) is a strategy that involves the use of fluids, packed red cells and inotropes to reach target hemodynamic parameters including cardiac output and oxygen delivery to prevent organ dysfunction [46,82,83]. Many high risk surgical patients (both elective and emergency) are admitted to the ICU in the perioperative period, often with many comorbidities and GDT in this time period has been associated with fewer complications (including AKI) and improved mortality [84]. In 2009, a meta-analysis demonstrated that AKI is significantly reduced by perioperative hemodynamic optimization, whether done in the pre-, intra- or postoperative period [46]. This is particularly relevant when resources for pre-operative optimization are limited. Of note, meeting physiological values was as ‘reno-protective’ as meeting supra-normal values, which itself may be associated with other complications although this remains a point of debate [46].

The use of sodium bicarbonate has been addressed in cardiac surgery. Following on from work supporting the use of urinary alkanization in contrast nephropathy, a pilot RCT in 100 post-CPB surgical patients showed a reduction in the incidence of AKI in patients receiving sodium bicarbonate versus a placebo saline infusion although no changes were demonstrated in either the need for RRT or mortality [85]. Further trials in this area are ongoing/promising.

### Avoidance of nephrotoxic agents

A number of different medications commonly used in the perioperative period have potentially harmful effects on renal function. Angiotensin-converting enzyme inhibitors (ACEi) and angiotensin receptor blockers (ARB) and NSAIDs are among drugs known to affect renal autoregulation. Whether or not to continue ACEi/ARB in the perioperative period remains under debate although a meta-analysis has shown ACEi confer no protective benefits in this time period [86].

NSAIDs can also cause interstitial nephritis and their association with the development of AKI led to recommendations from the Medicines and Healthcare Products Regulatory Agency suggesting that they should be avoided in all patients with hypovolemia and sepsis regardless of renal function [87]. Antibiotics can lead to AKI by either direct injury, for example aminoglycosides in high concentrations, thereby necessitating monitoring of drug levels, or secondary to an acute interstitial nephritis, for example penicillins, quinolones and cephalosporins.

The role of intravenous contrast in AKI is well recognized and where its use is unavoidable, the minimum possible dose should be given as well as using the newer iso-osmolar and low-osmolar non-ionic contrast, now recognized to be less toxic [88,89]. Surgery should be postponed in stable patients with contrast-induced AKI. Whether oral N-acetylcysteine confers any protective benefit in this situation remains controversial [90].

### Hemodilution and transfusion in cardiac surgery

Specific to cardiac surgery, the roles of hemodilution and transfusion have also been studied. There is a known association between AKI and erythrocyte transfusion in cardiac surgery [91-94]. More recently, a single center study has both confirmed this and suggested that the level of pre-operative anemia also has an impact, being associated with a more pronounced increase in the incidence of AKI [95]. There is, however, ongoing work into the role of erythropoietin (EPO) in this setting, with a small pilot trial confirming its effectiveness although a larger trial is required before this could be recommended [96]. Hemodilution is induced in the setting of cardio-pulmonary bypass surgery, in theory decreasing blood viscosity and improving microcirculatory flow in the presence of both hypoperfusion and hypothermia. However, this has been associated with a significant increase in the incidence of AKI and, as such, current guidelines underline the importance of limiting hemodilution, with the Society of Thoracic Surgeons and the Society of Cardiovascular Anaesthesiologists recommending maintenance of hematocrit >21% and hemoglobin >7 g/dl [91,97-100].

### Pharmacological interventions

There have been many attempts to find pharmacological interventions in the management of AKI, with the ongoing challenge regarding the use of standard definitions and end-points making it difficult to directly compare trial evidence. Until recently there have been no drugs that have consistently been demonstrated to confer benefit, although there is now some emerging evidence in the setting of cardiac surgery [80,86].

### Dopamine

Dopamine has been extensively used and its place debated over the years, with much of the early enthusiasm driven by the assumption that increased renal blood flow seen with low-dose dopamine is beneficial in the management of AKI [101-103]. A meta-analysis published in 2001, however, demonstrated no benefit using dopamine for either the prevention or treatment of AKI. This recommendation followed identification and analysis of 58 studies, 24 of which reported the outcomes reviewed (including 17 RCTs) [102]. This was further supported in a systematic literature review, last updated in 2008 [86].

### Fenoldopam

Fenoldopam is a selective DA-1 agonist which to date has had mixed results when used in the management of AKI [80,104,105]. In cardiac surgery, however, fenoldopam was shown to consistently reduce the need for RRT and mortality, although its use is potentially complicated by systemic hypotension [80,106]. This undesirable side effect may be improved with the use of intra-renal infusions, an innovative/emerging strategy which to date has proven successful in case reports although further trial information with a larger number of patients is required [105].

### Diuretics (furosemide/mannitol)

While use of diuretics may improve urine output in the setting of acute kidney injury, again there is no evidence to support that they confer any improvement in outcomes measured (including need for RRT and mortality) [80,101,107]. Furthermore, use of furosemide has been shown to be not only ineffective but also detrimental, associated with higher postoperative serum creatinine levels in cardiac patients [80,102,108]. Of note, mannitol is often added to the priming solution used in CPB surgery. Although initially shown to confer some preventive benefits in children undergoing CPB surgery, these results have not been reproduced in repeat studies, with a suggestion that mannitol is actually associated with increased tubular injury when given in combination with dopamine [81,106,108,109].

### Atrial natriuretic peptide (ANP)

ANP is produced by cardiac atria in response to atrial dilatation and its properties as an endogenous diuretic and natriuretic substance led to further evaluation of ANP as another potential therapy. Early RCTs showed a benefit in only a sub-group of oliguric patients which was not reproduced in follow up studies and systemic hypotension was noted to be a complicating factor [110-113]. A significant reduction in the need for RRT was, however, seen in post-cardiac surgical patients with decompensated congestive cardiac failure (CCF) who received low-dose infusions of recombinant human ANP. Of note, the lower dose infusion was associated with a decrease in the incidence of systemic hypotension, which in itself may contribute to the change in results seen [113,114]. Outside of cardiac surgery, there is at present no perceived benefit with ANP [80,114].

### Nesiritide (recombinant human β natriuretic peptide)

Nesiritide is another cardiac natriuretic peptide that is currently under evaluation. Initial results in both cardiac and abdominal aneurysm repair surgery have shown potential protective benefits with an overall reduction in mortality, however, there is a possible association with increased mortality in acutely decompensated heart failure [114-116]. Overall, nesiritide warrants further investigation before recommendations/conclusions can be confidently made [114-116].

### Theophylline

Theophylline, an adenosine antagonist, in theory is proposed to preserve renal blood flow by attenuating vasoconstriction of renal vessels [117,118]. Several small studies have been conducted using theophylline in contrast-induced nephropathy; however, a meta-analysis in 2005 was inconclusive and recommended that a RCT in this area with a defined hydration protocol would be of benefit [117]. In the setting of CPB surgery, an infusion of theophylline conferred no benefit in reducing the incidence of AKI [118].

### N-acetylcysteine

The role of N-acetlycysteine, an antioxidant most commonly used to enhance formation of glutathione after paracetamol overdose, has not been shown to confer any protective benefits in the perioperative period [119,120]. As mentioned above, there may be some role for this agent in contrast-induced nephropathy [90].

### Glycemic control

A landmark study in 2001 demonstrated tight glycemic control and showed improved outcomes in an Intensive Therapy Unit setting, with a 41% reduction in AKI requiring RRT [121]. This has, therefore, sparked renewed focus in this area; however, subsequent studies have not reproduced these benefits [122]. More recently, in cardiac surgery, while severe intraoperative and early postoperative hyperglycemia was associated with poorer outcomes (including an increased incidence of AKI), incremental decreases in mean glucose concentrations did not show consistent improvements in outcomes [123]. Given the inconsistent results seen, the concept of tight glycemic control needs further reassessment, with development of strategies that focus on avoiding large variations in blood glucose and hypoglycemia [122,123].

### Prophylactic RRT

There is currently insufficient evidence to support the use of prophylactic RRT in high-risk patients undergoing major surgery. Indeed it seems somewhat counterproductive to dialyze someone in order to prevent dialysis. A single center study in which 44 patients were randomized either to receive prophylactic dialysis or postoperative dialysis if indicated did,,however, show both a decrease in mortality (4.8% versus 30.4%) and in AKI requiring RRT [124]. An effect size this large is statistically very unlikely in practice. Similarly, in the setting of contrast nephropathy, a small single center RCT showed that prophylactic hemofiltration was associated with a decrease in both mortality and morbidity although these findings are limited by the lack of standardized hydration protocols and use of N-Acetyl Cysteine in this trial [125]. However, more evidence is required before this invasive strategy can be recommended.

### RRT in established AKI

Many of the goals of modern treatment are to prevent AKI; when established, however, RRT then plays an important role in the subsequent management, with approximately 15% of patients in intensive care with AKI receiving dialysis [126]. Despite decades of research and debate, this remains an area where there is no clear consensus as to the optimal timing, modality or dose of RRT, yet it is recognized as a significant factor affecting outcome in critically ill patients [1,11,127,128]. Earlier studies suggested a benefit to higher dose hemodialysis or hemofiltration [127] although this was not confirmed in follow up studies and the debate rages on [128-131].

## Conclusions

AKI is a serious and often under-appreciated complication in the perioperative period, with even small rises in serum creatinine associated with both increased morbidity and mortality. The use of risk stratification indices should help in the identification of high risk patients, useful both for clinical practice and on-going research. Recent advances have been made in the field of biomarkers although this work has yet to be translated into clinical practice and the mainstay of treatment remains preventive, aiming to keep patients optimally hydrated while avoiding nephrotoxic agents. There are no pharmacological agents yet with proven benefits in the management of AKI although there is some emerging evidence favoring the use of fenoldopam and ANP in the setting of cardiac surgery, with novel techniques in the delivery of agents helping to overcome systemic side effects.

## Abbreviations

ACEi, Angiotensin converting enzyme inhibitor; AKI, Acute kidney injury; AKIN, Acute Kidney Injury Network; ANP, Atrial natriuretic peptide; ARB, Angiotensin receptor blocker; ARDS, Acute respiratory distress syndrome; AUCROC, Area under the receiver operating characteristic curve; CABG, Coronary artery bypass graft; CCF, Congestive cardiac failure; CKD, Chronic kidney disease; CPB, Cardiopulmonary bypass; DIC, Disseminated intravascular coagulation; EF, Ejection fraction; EPO, Erythropoietin; GDT, Goal directed therapy; GFR, Glomerular filtration rate; IABP, Intra-aortic balloon pump; Il-18, Interleukin-18; KDIGO, Kidney Disease Improving Global Outcomes; KIM-1, Kidney injury molecule-1; LVF, Left ventricular function; MACE, Major adverse cardiac events; NGAL, Neutrophil gelatinase-associated lipocalin; NSAIDs, Non-steroidal anti-inflammatory drugs; RIFLE, Risk Injury, Failure, Loss and End-stage; RCT, Randomized controlled trial; RRT, Renal replacement therapy; TTP, Thrombotic thrombocytopenic purpura.

## Competing interests

The authors declare that they have no competing interests.

## Authors' contributions

SC wrote the first draft. SC and AS edited and finalized the review. Both authors read and approved the final manuscript.
